# Validation of the english assessment of criteria for specific internet-use disorders (ACSID-11) for tinder and online pornography use

**DOI:** 10.3389/fpsyt.2025.1595502

**Published:** 2025-07-16

**Authors:** Magdalena Liberacka-Dwojak, Germano Vera Cruz, Monika Wiłkość-Dębczyńska, Lucien Rochat, Riaz Khan, Silke M. Mueller, Matthias Brand, Yasser Khazaal

**Affiliations:** ^1^ Department of Psychology, Kazimierz Wielki University, Bydgoszcz, Poland; ^2^ Department of Psychology, University of Picardie Jules Verne, Amiens, France; ^3^ Department of Mental Health and Psychiatry, Specialized Facility in Behavioral Addiction ReConnecte, University Hospitals of Geneva, Geneva, Switzerland; ^4^ Department of Mental Health and Psychiatry, Frontier Medical College, Abbottabad, Pakistan; ^5^ General Psychology: Cognition and Center for Behavioral Addiction Research (CeBAR), University of Duisburg-Essen, Duisburg, Germany; ^6^ Addiction Medicine, Department of Psychiatry, Lausanne University Hospital, Lausanne, Switzerland

**Keywords:** dating applications-use disorder, online pornography-use disorder, behavioral addictions, diagnostic criteria, ICD-11, validation

## Abstract

**Background and aims:**

Internet behaviors, like social networking, dating apps, or online pornography, may develop into disorders due to their addictive potential, aligning with other specific Internet-use disorders in ICD-11. This study aimed to validate the Assessment of Criteria for Specific Internet-use Disorders (ACSID-11) for Tinder and online pornography use in English-speaking respondents.

**Methods:**

The ACSID-11 was administered to active Tinder and pornography users fluent in English (N = 1823) alongside the Problematic Tinder Use Scale (PTUS), Short Problematic Pornography Consumption Scale (PPCS-6), and Sexual Desire Inventory-2 (SDI-2). Confirmatory factor analyses (CFA) examined ACSID-11’s factor structure, and correlation analyses assessed its convergent validity. The eight-factor structure was confirmed and was superior to other tested solutions both for Tinder and online pornography use. ACSID-11 scores correlated with the PTUS, PPCS-6, and SDI-2.

**Discussion and Conclusions:**

The ACSID-11 is a suitable tool for testing other specified disorders due to addictive behaviors such as Tinder and online pornography-use disorders, based on ICD-11 diagnostic criteria, valuable in assisting with a detailed clinical diagnosis. Moreover, the tool is also valid for English-speaking respondents

## 1 Introduction

Online services seem particularly appealing and offer many benefits due to their widespread use and easy access to the Internet. Beyond many advantages for the majority of people, some individuals’ online behaviors could develop into an uncontrolled addictive form, occurring as a public health issue (1, 2). Addressing this issue the 5^th^ revision of the *Diagnostic and Statistical Manual of Mental Disorders* (3) (DSM-5) recognized the *Internet gaming disorder* as a ‘conditions for further study’, while the 11^th^ revision of the *International Classification of Diseases* (4) included gaming disorder (6C51) as a diagnosis in the *Disorders due to addictive behaviors* section.

It seems that very specific online behavior may become a health problem that can be considered as a global challenge (2, 5). An increasing amount of studies supports the hypothesis that other specific Internet behaviors, such as the use of social networking sites, including dating apps, or online pornography may also develop into a disorder due to its potentially addictive form, comparable to gaming (2, 6, 7).

Accordingly, it has been also proposed that both social-networks-use and online pornography-use disorders might be included within the category 6C5Y: *Other* sp*ecified disorders due to addictive behaviors* in ICD-11 (4). Nonetheless, there is still not enough empirical evidence regarding their specific features. Theoretical frameworks assume that similar psychological processes cause different types of online addictive behaviors, as a result of individual susceptibility, psychopathological factors, and cognitive and affective factor interaction (8, 9).

The key features of online addictive behaviors identified in research and captured in ICD-11 criteria for gaming and gambling disorders include: impaired control over the use, increasing priority given to the use, and continuation or escalation of use despite negative consequences (7, 10, 11). Moreover, the behavioral pattern leads to functional impairment in important areas of daily life and/or marked distress (4).

Behaviors such as online pornography (11, 12), social networking sites, and online communication apps (7, 13), as well as dating apps use (13–15) are characterized by diminished control over its consumption with potentially clinically relevant phenomena, comparable to other addictive behaviors (2). In light of these commonalities, scales for dating applications and online pornography use were included as two distinct digital services in our study. However, the usage profiles of both services can be different, so it seems highly relevant to use them separately.

Such conditions are candidates for the International Classification of Diseases (ICD-11) designation of “other specified disorders due to addictive behaviors” (2, 6, 11). More specifically, dating apps are seen as a specific form of social networking that allows for searching for a life or sexual partner (14, 16). Thus, the negative consequences of poorly controlled use can be significant and linked to functional impairment. It is believed that the key mechanisms involved in the pathological use of both online pornography and social networking sites (i.e. dating apps) are comparable with those involved in gaming disorder (6) although the causality of affective and cognitive mechanisms and the potential specificity of certain applications is not yet clear (17).

Establishing criteria and validating assessment tools for impaired Internet-related behaviors could be helpful for research and clinical practice, as there is a growing number of online services. Most of the available questionnaires designed to screen for such addictive behaviors are based on the components model by Griffiths (18), for instance, The Bergen Social Media Addiction Scale (19). Different scales were furthermore developed for different behaviors such as gaming (20), porn (11), and Tinder use (13). Each of these scales are based on six components, such as salience, mood modification, tolerance, withdrawal symptoms, conflict, and relapse. However, the Griffith’s model has been widely criticized, as the use substance addiction criteria (e.g. salience, tolerance) to operationalize and assess behavioral addictions is believed not to be valid (21). In the light of this criticism, it becomes significantly relevant to provide new, valid scales to assess the behavioral addictions.

To explicitly address this research gap, the present study focuses on the development and validation of assessment instruments grounded in the ICD-11 conceptualization of behavioral addictions. Existing tools often remain tied to substance-use frameworks, which do not fully reflect the specific mechanisms, trajectories, and impacts of behavioral addictions. Moreover, current research frequently suffers from methodological shortcomings, including limited cross-cultural validation, and inconsistencies in measurement approaches across different behaviors. Importantly, while gaming disorder has received increasing empirical attention, other forms of behavioral addiction—such as compulsive use of pornography or dating applications—remain insufficiently studied, despite their growing prevalence and potential for significant psychosocial consequences. The Assessment of Criteria for Specific Internet-use Disorders (ASCID-11) responds to these challenges by translating ICD-11 diagnostic criteria into a behavior-general, domain-flexible tool. Designed to be applicable across diverse online behaviors, ASCID-11 offers a unified and conceptually robust framework for advancing research on a broad spectrum of problematic Internet-use behaviors (2).

The initial validation study of the ACSID-11, conducted on 985 active, German Internet users, indicated that the four-factorial structure (based on the ICD-11 criteria) is valid for the assessment of multiple types of problematic internet use. While the original validation study demonstrated promising psychometric properties, its external validity still needs further investigations. The original sample included German-speaking Internet users from the general (non-clinical) population. Since then, validation studies have been conducted in various countries and languages, some of which have already been published (e.g., Thai: 22; Chinese: 23; Traditional Chinese: 24). Then, the instrument initially focused on specific Internet-use behaviors (i.e., gaming, shopping, pornography use, and social network use), but not on emerging domains like dating apps. In the present study, we aimed to examine the scale’s psychometric validity and factorial structure across distinct domains of digital behavior, in line with the ICD-11 framework in an English-speaking sample. This approach allows us to assess whether a single measurement tool can validly capture the core features of various behavioral addictions, thereby contributing to a more unified and conceptually consistent psychometric model for Internet-use disorders beyond gaming.

## 2 Methods

### 2.1 Participants

A total of 1823 people aged from 19 to 65 years (M = 31.67; SD = 6.75) participated in the survey. There were no missing data. Gender was distributed for 48.51% for males, 50.75% for females, and 0.90% for non-binary. The participants were citizens of the following countries: the United Kingdom (77.2%), the United States (17.8%), Ireland (1.6%), Australia (1.5%), Sweden (1.3%), and New Zealand (0.6%). The respondents’ nationalities differentiated between 27 different European countries (76.1%), North American countries (16%), Asian countries (2.7%), African countries (2.0%), Ocean countries (1.8%), Latino-America (0.8%), and Middle east countries (0.8%). 49.40% of participants declared to be single, while 48.50% reported being in a relationship (31.10% in an informal relationship, 17.40% married). 2.00% were divorced and 0.10% widowed. Additionally, 26.50% of respondents declared having a low socio-economic level, 69.40% an intermediate level, and 4.10% a high level.

### 2.2 Recruitment and sampling

The participants were anonymously recruited via the online crowdsourcing platform Prolific (25). The data were collected from 01.11.2022 to 11.11.2022. To be invited, participants have to be adults (older than 18, English fluent, and reporting porn and Tinder use in the last six months). Prolific has grown significantly in the last years due to its various advantages, such as exclusive dedication to research studies or an ethnically and geographical variety of participants. Moreover, participants seem to be more naïve to experimental research tasks. It offers good recruitment standards and good quality of data for research purposes (26, 27). We aimed to investigate individuals using both porn and Tinder to ensure that the sample represented a broader spectrum of online behaviors that might share common psychological processes to help in establishing the tool’s applicability.

### 2.3 Data collection material

The data was gathered anonymously through an online survey with the following instruments:

Socio-demographic questions included age, sex, marital status, level of education, socio-economic status, and the average time spent on Tinder/pornography during the typical week (hours per week) in the last month.

*Assessment of Criteria for Specific Internet-use Disorders* (ACSID-11) (2) is a questionnaire that is a new 11-items screening tool acquiring ICD-11 criteria for potential Internet-use disorders, including online pornography and social network use. It measures the three main criteria (with three items each), *Impaired control* (IC), *Increased priority given to the online activity* (IP), *Continuation/escalation* (CE) of Internet use despite negative consequences. Two additional items assess *Functional impairment in daily life* (FI) and *Marked distress* (MD) due to the online activity. The scale assesses the frequency and intensity of each symptom using a two-part response format. Participants indicate, per item for each activity, how often and how intense each experience was in the last 12 months on a four-point Likert scale, where 0 corresponds to ‘never/not at all intense’, and 3 to ‘often/intense’. In the current study, respondents were requested to answer all questions regarding two subtypes of Internet use: Tinder use and pornography use, specifically the questionnaire introduction related to the “Tinder and pornography activity”. The ACSID-11 includes functional impairment as a core component, as suggested to reduce risk of over pathologization observed in relation to the scales based on the component model of addiction. In this context, ACSID-11 offers several advantages, like alignment with ICD-11, and comprehensive assessment by incorporating both frequency and intensity dimensions. Furthermore, the scale is not based on the component model. The original version was validated among the German-speaking participants screened for gaming disorder, online buying-shopping disorder, online pornography-use disorder, social networking sites-use disorder, and online gambling disorder. The English version of the tool was proposed in the original article (2). In the present study, the original English version provided by the authors was used. Although the English version of the tool was introduced in the original publication (2), it has not yet undergone psychometric validation in an English-speaking sample.*Problematic Tinder Use Scale* (PTUS; (13) is a 6-item scale based on Griffiths’ concept of problematic use that measures the six core elements of problematic Tinder use in terms of salience, tolerance, mood modification, relapse, withdrawal, and conflict. The reliability of the scale was α = 0.869.

*Short Problematic Pornography Consumption Scale* (PPCS-6; (11) is a shorter version of the Problematic Pornography Consumption Scale that assesses problematic pornography use via Griffiths’ six-component addiction model with six elements coherent with PTUS. The reliability of the scale was α = 0.875.

*Sexual Desire Inventory-2* (SDI-2; (28) is a self-reported 14-item inventory measuring sexual desire in men and women. It categorizes sexual desire into two dimensions: dyadic and solitary, assessing the strength, frequency, and importance of an individual’s desire for sexual activity with others and by themselves (29). The reliability of the scale was α = 0.875.

### 2.4 Ethics

The survey was conducted in compliance with the Swiss Human Research Act. Respondents provided digital informed consent for the study contribution. The participation was voluntary and all data was gathered anonymously. The participants received financial compensation according to the Prolific standards. All participants were restricted to be ≥ 18 years. The ethical approval no. KB 390/2022 was obtained from The Bioethics Committee.

### 2.5 Data analysis

A descriptive analysis of the characteristics of ACSID-11 responses and ACSID-11, PTUS, PPCS-6, and SDI-2 factors was conducted. The reliability was measured using Cronbach’s alpha (α) and Guttman’s Lambda (λ2) with coefficients > 0.7 indicating acceptable internal consistency (30). Pearson correlation analyses were used to assess the convergent validity between different measures of the same or related constructs. Confirmatory factors analysis (CFA) was handled to test the construct validity of the ACSID-11. The model was determined by the following indices: root mean square error of approximation (RMSEA), comparative fit index (CFI), goodness of fit index (GFI), adjusted goodness of fit (AGFI), normed fit index (NFI), and Tucker-Lewis index (TLI). It was assumed that the ACSID-11 would be considered valid if the model fit indices will be as presented: CFI, GFI, AGFI, NFI, and TLI are > 0.9; RMSEA < 0.08; SRMR < 0.08; χ2/df < 5 (31). All analyses were performed using SPSS v27 and Python with pandas, semopy, and graphviz libraries using the generalized least squares estimation.

Although the primary goal of the study was confirmatory—to test predefined ICD-11-based structures using CFA—we conducted an exploratory factor analysis (EFA) as an initial step to examine whether the data would empirically support alternative factor configurations. The number of factors retained in the EFA was determined using the Kaiser criterion (eigenvalues > 1) and visual inspection of the scree plot. This approach was justified by the novelty of applying the ACSID-11 in English and to domains such as dating app and online pornography use, which had not been previously validated.

## 3 Results

### 3.1 Descriptive statistics

Regarding both Tinder and online pornography use, all ACSID-11 items assessing frequency and intensity ranged between 0 and 3 (see **Tables 1**, **2**). Relatedly to the original version, all items had relatively low mean values and were right-skewed. Kurtosis was especially high for all items regarding *Continuation/Escalation* (CE1-CE3) and the *Increased priority given to the online activity* third item (IP3) for the intensity of Tinder use. The mean ACSID-11 factor values were highest for the *Impaired control* (IC) in the frequency and intensity of both Tinder and online pornography use.

**Table 1:** Descriptive statistics of the ACSID-11 items measuring Tinder use.

Item	Min	Max	M	(SD)	Skewness	Kurtosis
Frequency factor						
IC1	0	3	0.759	0.827	0.823	-0.121
IC2	0	3	0.614	0.931	1.304	0.460
IC3	0	3	0.411	0.791	1.903	2.655
IP1	0	3	0.355	0.686	1.972	3.265
IP2	0	3	0.287	0.648	2.342	4.862
IP3	0	3	0.227	0.556	2.559	6.076
CE1	0	3	0.235	0.584	2.633	6.551
CE2	0	3	0.202	0.531	2.818	7.769
CE3	0	3	0.208	0.535	2.746	7.374
FI1	0	3	0.355	0.654	1.811	2.594
MDI1	0	3	0.301	0.628	2.189	4.403
Intensity factor						
IC1	0	3	0.563	0.788	1.272	0.826
IC2	0	3	0.519	0.836	1.499	1.236
IC3	0	3	0.372	0.746	2.040	3.343
IP1	0	3	0.321	0.660	2.110	3.853
IP2	0	3	0.264	0.630	2.537	5.995
IP3	0	3	0.197	0.542	3.043	9.403
CE1	0	3	0.222	0.584	2.891	8.238
CE2	0	3	0.172	0.513	3.352	11.653
CE3	0	3	0.189	0.522	3.009	9.105
FI1	0	3	0.312	0.650	2.193	4.371
MDI1	0	3	0.295	0.646	2.330	4.995

N, 1823. IC, impaired control; IP, increased priority; CE, continuation/escalation; FI, functional impairment; MD, marked distress.

**Table 2:** Descriptive statistics of the ACSID-11 items measuring online pornography use.

Item	Min	Max	M	(SD)	Skewness	Kurtosis
Frequency factor						
IC1	0	3	1.307	0.891	0.277	-0.639
IC2	0	3	1.091	1.024	0.479	-0.964
IC3	0	3	0.801	1.001	0.935	-0.397
IP1	0	3	0.676	0.853	1.064	0.224
IP2	0	3	0.524	0.810	1.448	1.205
IP3	0	3	0.443	0.761	1.661	1.909
CE1	0	3	0.372	0.721	1.970	3.156
CE2	0	3	0.351	0.691	1.980	3.187
CE3	0	3	0.361	0.714	2.035	3.469
FI1	0	3	0.556	0.785	1.330	1.073
MDI1	0	3	0.385	0.714	1.887	2.910
Intensity factor						
IC1	0	3	1.155	0.942	0.369	-0.802
IC2	0	3	0.967	1.011	0.647	-0.793
IC3	0	3	0.749	0.988	1.022	-0.244
IP1	0	3	0.624	0.865	1.187	0.369
IP2	0	3	0.485	0.808	1.602	1.648
IP3	0	3	0.425	0.777	1.802	2.344
CE1	0	3	0.346	0.724	2.168	3.970
CE2	0	3	0.308	0.668	2.241	4.378
CE3	0	3	0.330	0.705	2.214	4.184
FI1	0	3	0.480	0.779	1.563	1.615
MDI1	0	3	0.368	0.728	1.980	3.087

N, 1823. IC, impaired control; IP, increased priority; CE, continuation/escalation; FI, functional impairment; MD, marked distress.

The respondents claimed to spend average 4 hours per week both on online pornography and 3.50 hours on Tinder. The mean score for the *Problematic Tinder Use Scale* use was 1.682 (SD = 0.749) and for the *Short Problematic Pornography Consumption Scale* 2.805 (SD = 1.355). The mean score for sexual desire was the highest for the dyadic desire (M = 5.389; SD = 1.267) and lowest for the solitary desire (M = 5.039; SD = 1.567) (see **Table 3**).

**Table 3:** Descriptive statistics of the factor and overall scores of the ACSID-11, problematic tinder use scale, short problematic pornography consumption scale, and sexual desire inventory, and average time spent on tinder/online pornography (hours per week).

	Tinder use				Online pornography use			
	Min	Max	M	(SD)	Min	Max	M	(SD)
ACSID-11: frequency								
ACSID-11_IC	0	3	0.595	0.697	0	3	1.066	0.810
ACSID-11_IP	0	3	0.289	0.546	0	3	0.548	0.708
ACSID-11_CE	0	3	0.215	0.460	0	3	0.361	0.591
ACSID-11_FI	0	3	0.328	0.570	0	3	0.470	0.678
ACSID-11_total	0	2.63	0.357	0.478	0	3	0.612	0.594
ACSID-11: intensity								
ACSID-11_IC	0	3	0.485	0.651	0	3	0.957	0.831
ACSID-11_IP	0	3	0.261	0.532	0	3	0.510	0.721
ACSID-11_CE	0	3	0.194	0.459	0	3	0.328	0.587
ACSID-11_FI	0	3	0.303	0.574	0	3	0.424	0.683
ACSID-11_total	0	3	0.311	0.468	0	3	0.555	0.609
Average hours per week spent on Tinder	2	12	3.500	1.800				
PTUS	1	4.83	1.682	0.749				
Average hours per week spent on online pornography					2	12	4.000	1.842
PPCS-6					1	7	2.805	1.355
SDI-2								
Dyadic					1	8	5.389	1.267
Solitary					1	8	5.039	1.567

N, 1823. IC, impaired control; IP, increased priority; CE, continuation/escalation; FI, functional impairment; MD, marked distress; PTUS, Problematic Tinder Use Scale; PPCS-6, Problematic Pornography Consumption Scale; SDI-2, Sexual Desire Inventory. The ‘Max’ values reflect the highest observed mean score in the sample for each factor or total scale. Theoretical maximum for any mean-based ACSID-11 score is 3.00.

### 3.2 Reliability

Reliability statistics are shown in **Table 4**. ACSID-11 both for Tinder and online pornography use reliability was acceptable as Cronbach’s alpha and Guttman’s Lambda were above the desired threshold (α > 0.7; λ2 > 0.7). The results also indicated the acceptable reliability of the PTUS, PPCS-6, and SDI-2.

**Table 4:** Reliability statistics of ACSID-11 for Tinder and online pornography use.

	Tinder use		Online pornography use	
	α	λ2	α	λ2
ACSID-11: frequency				
ACSID-11_IC	0.754	0.759	0.778	0.802
ACSID-11_IP	0.828	0.829	0.848	0.848
ACSID-11_CE	0.785	0.785	0.781	0.782
ACSID-11_FI	0.737	0.737	0.981	0.918
ACSID-11: intensity				
ACSID-11_IC	0.762	0.765	0.804	0.809
ACSID-11_IP	0.837	0.837	0.856	0.857
ACSID-11_CE	0.808	0.810	0.790	0.791
ACSID-11_FI	0.727	0.727	0.913	0.913
ACSID-11: total score				
	α		λ2	
PTUS	0.875		0.878	
PPCS-6	0.869		0.872	
SDI-2	0.875		0.880	

### 3.3 Confirmatory factor analysis

Five distinct models were tested to examine the factor structure of online pornography and Tinder use, as outlined in the Exploratory Factor Analysis (EFA) and Confirmatory Factor Analysis (CFA) procedures (see **Table 5**). To test the factorial structure of the ACSID-11 across Tinder and online pornography use, we examined five models: two-factor, three-factor, four-factor, eight-factor, and second-order eight-factor structures. The two- and three-factor models were derived from the results of an exploratory factor analysis (EFA), which was conducted using the Kaiser criterion and scree plot inspection as guides for factor retention. In turn, the four-factor and eight-factor solutions were theory-driven and constructed based on ICD-11 diagnostic domains. This approach allowed us to compare empirical structures with conceptually predefined models and assess the scale’s capacity to represent clinically relevant dimensions of Internet-use disorders.

**Table 5:** Fit indices of the tested models for Tinder and online pornography measured by ACSID-11.

Model	df	CFI	GFI	AGFI	NFI	TLI	RMSEA
Online pornography							
Two-factors model	197	0.942	0.937	0.926	0.937	0.932	0.080
Three-factors model	195	0.952	0.948	0.938	0.948	0.944	0.072
Four-factors model	192	0.954	0.950	0.939	0.950	0.945	0.072
Second-order model	189	0.967	0.952	0.942	0.952	0.947	0.070
Eight-factors model	170	0.985	0.981	0.974	0.981	0.980	0.043
Tinder use							
Two-factors model	197	0.931	0.926	0.913	0.926	0.919	0.082
Three-factors model	195	0.938	0.933	0.921	0.933	0.927	0.078
Four-factors model	192	0.943	0.938	0.925	0.938	0.931	0.075
Second-order model	189	0.952	0.948	0.936	0.946	0.942	0.069
Eight-factors model	170	0.976	0.972	0.961	0.972	0.968	0.052

The two-factor model was created based on EFA for both pornography and Tinder use. For both, the first factor included items related to impaired control, combining frequency and intensity ratings. The second factor covered increased priority, continuation/escalation, and functional impairment, combining frequency and intensity ratings.

The three-factor model was similarly derived from EFA for both pornography and Tinder use. For pornography use, the first factor included items related to impaired control, combining frequency and intensity ratings. The second factor included items related to increased priority, combining frequency and intensity ratings. The third factor comprised items related to continuation/escalation and functional impairment, including frequency and intensity ratings. For Tinder use, the first factor included items related to impaired control for frequency and intensity ratings. The second factor consisted of items related to increased priority for frequency and intensity ratings, as well as two items from continuation/escalation (ce1 and ce2) for frequency and intensity ratings. The third factor comprised one item from continuation/escalation (ce3) for frequency and intensity ratings, along with items related to functional impairment for frequency and intensity ratings.

The four-factor model combined frequency and intensity items into four factors without detailing the specific items in each factor. These factors included impaired control, increased priority, continuation/escalation, and functional impairment.

The eight-factor model separated frequency and intensity items into distinct factors, resulting in eight factors. These factors included frequency and intensity impaired control, frequency and intensity increased priority, frequency and intensity continuation/escalation, frequency and intensity functional impairment.

Finally, the second-order eight-factor model introduced higher-order factors combining the eight lower-order factors. This model consisted of two higher-order factors (Frequency and Intensity) and eight first-order factors. Both factors comprised impaired control, increased priority, continuation/escalation, and functional impairment.

**Figure 1:**
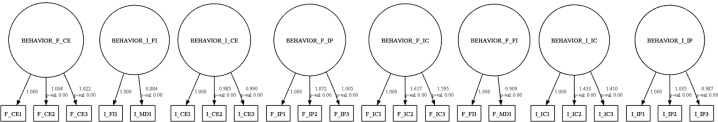
ACSID-11 model structure and factor loadings for online pornography use.

**Figure 2:**
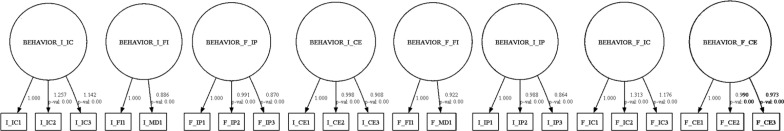
ACSID-11 model structure and factor loadings for Tinder use.

As presented in the **Table 5**, the eight-factor model indicated the best fit. The factor loadings are shown in **Figure 1** (online pornography use) and **Figure 2** (Tinder use).

### 3.4 Correlation analysis

Correlations between ACSID-11, PTUS, and average time spent on Tinder were analyzed to measure the construct validity of the ACSID-11 for Tinder usage (see **Table 6**). The ACSID-11 frequency and intensity total scores correlated positively with both scales with medium to large effect sizes (r = {0.360; 0.877}) with the highest scores for the PTUS and ACSID-11. Relations between ACSID-11, PPCS-6, SDI-2, and average time spent on online pornography were tested to measure the construct validity of the ACSID-11 for online pornography usage (see **Table 7**). The ACSID-11 frequency and intensity scores correlated positively with small to medium effect size (r = {0.131; 0.913}). The highest relations were found for the PPCS-6 and ACSID-11. Moreover, detailed correlations between online pornography and Tinder use were computed to show the convergent validity (see **Table 8**) The moderate correlations between these two behaviors (r = {0.243; 0.631}) confirm the convergent validity of the tool between related dimensions of behavioral addiction in Tinder use and online pornography use. This indicates that the ACSID-11 is a reliable tool for assessing similar addictive behaviors across different internet-use contexts.

**Table 6:** Correlations between detailed ACSID-11 scores (frequency and intensity), average time spent on tinder, and problematic tinder Use.

	1)	2)	3)	4)	5)	6)	7)	8)	9)	10)
1) Frequency: Impaired control	1.000									
2) Frequency: Increased priority	0.609	1.000								
3) Frequency: Continuation/Escalation	0.549	0.722	1.000							
4) Frequency: Functional impairment	0.528	0.606	0.686	1.000						
5) Intensity: Impaired control	0.877	0.621	0.555	0.516	1.000					
6) Intensity: Increased priority	0.586	0.882	0.706	0.576	0.643	1.000				
7) Intensity: Continuation/Escalation	0.514	0.670	0.874	0.636	0.565	0.740	1.000			
8) Intensity: Functional impairment	0.492	0.582	0.642	0.863	0.521	0.614	0.655	1.000		
9) Average hours per week spent on Tinder	0.505	0.433	0.371	0.360	0.495	0.413	0.363	0.354	1.000	
10) Problematic Tinder Use	0.649	0.648	0.613	0.627	0.642	0.619	0.576	0.590	0.530	1.000

*all p-values were <0.001. Red text indicates p-values <0.001.

**Table 7:** Correlations between detailed ACSID-11 scores (frequency and intensity), average time spent on online pornography, oroblematic pornography consumption, and sexual desire (dyadic, solitary).

	1)	2)	3)	4)	5)	6)	7)	8)	9)	10)	11)	12)
1) Frequency: Impaired control	1.000											
2) Frequency: Increased priority	0.604	1.000										
3) Frequency: Continuation/Escalation	0.524	0.704	1.000									
4) Frequency: Functional impairment	0.584	0.680	0.736	1.000								
5) Intensity: Impaired control	0.875	0.625	0.540	0.588	1.000							
6) Intensity: Increased priority	0.575	0.909	0.678	0.666	0.646	1.000						
7) Intensity: Continuation/Escalation	0.497	0.686	0.913	0.713	0.560	0.717	1.000					
8) Intensity: Functional impairment	0.554	0.648	0.715	0.887	0.603	0.678	0.748	1.000				
9) Average hours per week spent on pornography	0.418	0.427	0.319	0.366	0.419	0.413	0.321	0.364	1.000			
10) Problematic Pornography Consumption	0.603	0.649	0.501	0.562	0.628	0.641	0.503	0.565	0.470	1.000		
11) Sexual desire (dyadic)	0.217	0.179	0.131	0.134	0.223	0.191	0.137	0.137	0.180	0.321	1.000	
12) Sexual desire (solitary)	0.320	0.301	0.198	0.248	0.327	0.296	0.207	0.231	0.332	0.499	0.397	1.000

*all p-values were <0.001. Red text indicates p-values <0.001.

**Table 8:** Correlations between detailed ACSID-11 scores for online pornography and Tinder use.

	Pornography use							
Tinder use	1)	2)	3)	4)	5)	6)	7)	8)
1) Frequency: Impaired control	0.467	0.438	0.288	0.274	0.264	0.243	0.227	0.219
2) Frequency: Increased priority	0.446	0.476	0.304	0.332	0.283	0.285	0.242	0.271
3) Frequency: Continuation/Escalation	0.392	0.394	0.541	0.519	0.456	0.423	0.352	0.340
4) Frequency: Functional impairment	0.388	0.417	0.521	0.558	0.444	0.464	0.340	0.382
5) Intensity: Impaired control	0.388	0.406	0.501	0.508	0.631	0.591	0.431	0.435
6) Intensity: Increased priority	0.365	0.409	0.475	0.524	0.588	0.611	0.406	0.442
7) Intensity: Continuation/Escalation	0.351	0.357	0.414	0.409	0.467	0.436	0.435	0.423
8) Intensity: Functional impairment	0.335	0.368	0.392	0.422	0.434	0.452	0.408	0.454

*all p-values were <0.001. Red text indicates p-values <0.001.

## 4 Discussion

This study aimed to validate the ACSID-11 for Tinder and online pornography use among English-speaking participants. The results indicate that ACSID-11 is suitable to capture ICD-11 criteria for specified disorders due to addictive behaviors. Positive correlations with the PTUS, PPCS-6, and SDI-2 indicated the construct validity of the tool.

The eight-factor model representing the ICD-11 criteria (1) *Impaired control*, (2) *Increased priority*, (3) *Continuation/escalation despite negative consequences*, and (4) *Functional impairment and marked distress* for frequency and intensity was confirmed. The eight-factor model showed superior fit compared to other tested solutions. Still, the second-order model also indicated a good fit.(see **Table 5**). Such eight-factor structure provides a comprehensive framework that aligns with the ICD-11 criteria for addictive behaviors. Each factor represents a critical aspect of the disorder, ensuring that the diagnosis captures the full scope of problematic behaviors. Moreover, the eight-factor model enables differentiation between various aspects of Internet-use disorders, which can be crucial for tailored interventions. Such an approach also allows for capturing clinically relevant symptoms, such as very frequent behavior with low intensity or the reverse. However, recently the paper by Oelker et al. (32) proposed a theoretically derived dichotomized scoring, which can be investigated in further studies, as it is claimed to address the issues of high inter-factor correlations and allows each factor to be represented by a dichotomous value, which may have high value for use in clinical practice. The results in this research are indeed comparable to those reported by Oelker et al. (32). The original model proposed by Müller et al. (2), although coherent with the results, computed two distinct models separately for frequency and intensity, which is an approach that may require further exploration.

The reliability of ACSID-11 was high for both behaviors, achieving similar results as the original version (2). The internal consistency was also good for other behaviors measured by PTUS, PPCS-6, and SDI-2 (see **Table 4**). Moreover, the ACSID-11 indicated also the convergent validity (see **Table 8**). It can be concluded that the response format is suitable for an assessment of different behavioral addictions in English-speaking respondents. The average results for the subscales for Tinder use are lower than for the pornography use (Paired Sample T-Tests indicate that all pairs showed significant differences between both the frequency and intensity measures, as indicated by the t-values and p-values that in all cases were <0.001.). It can be hypothesized that the prevalence of problematic use of online pornography is higher than for Tinder because it is more likely to be perceived by users as a behavioral addiction than social networking, which may be perceived as a typical part of social life or a way of finding a partner (33). However, the sampling biases could contribute to the variations in the results. Nonetheless, dating apps offer a quick and effective reward as users can receive positive social feedback, which extends their time on the app. Furthermore, it may be challenging to stop swiping due to the variety of potential relationships. This pattern is likely to contribute to individuals’ frequent use of online dating services, which might lead to problematic use (14). Moreover, providing clear cutoff scores to determine if the results are sufficient for diagnosing behavioral addictions would be beneficial. For the ACSID-11, only a proposed criteria-based cut-off exists so far (32), a clinical validation is still ongoing. Establishing thresholds requires validation against structured clinical interviews or diagnostic tools. Future studies may employ methods such as ROC curve analyses to define sensitivity and specificity-based cut-offs for screening purposes. Furthermore, future studies could examine different ways of applying the ACSID-11, including total scores, criterion-level analyses, or multi-criteria threshold approaches—such as the one recently proposed by Oelker et al. (32)—and compare their diagnostic performance against structured clinical interviews. Such investigations could enhance our understanding of how best to operationalize ICD-11 criteria across diverse behavioral addiction profiles and improve the scale’s clinical utility.

Construct validity of ACSID-11 for Tinder use was indicated by medium to large positive correlations with PTUS and average time spent on Tinder (see **Table 6**). For online pornography use, medium to large positive correlations between ACSID-11, PPCS-6, and average time spent on pornography were found (see **Table 7**). Moreover, small to medium positive relations were indicated between ACSID-11 for online pornography and SDI-2, achieving higher results for solitary sexual desire. This result is consistent with previous findings on associations between higher sexual desire and pornography use (34–36), as it may be a gateway to the fulfillment of sexual needs (37). Moreover, the neural responses to sexual stimuli are known to be related to sexual desire levels (38) that may be associated with the probability of developing problematic pornography use by increased motivational salience of sexual rewards (39). Such a diagnosis may be more appropriate for individuals exclusively suffering from poorly controlled pornography viewing, not accompanied by other non-pornography-related compulsive sexual behaviors (6). Moreover, the convergent validity results indicated that the ACSID-11 measures constructs consistently across different forms of internet-use behaviors, supporting its convergent validity.

The eight-factor model of ACSID-11 in English-speaking respondents indicated a good fit. It is also worth noticing that the sample contained English-speaking individuals with different nationalities (76.1% European, 16% North American, 2.7% Asian, 2.0% African, 1.8% Ocean, 0.8% Latino-america, and 0.8% Middle Eastern countries) with possible cultural differences and include people from different age groups, including seniors which is in adequation with recent studies on porn use among people older than 65 (40) and on marketing reports on dating apps (41). The original study included active Internet users from the German-speaking area but the authors did not provide information about the participants’ nationality (2). As German-speaking areas consist of many nationalities, it can be assumed that both German and English versions of ACSID-11 are suitable to capture Internet-related addictive behaviors for English and/or respectively German fluent people independently of cultural background.

In order to define the diagnostic criteria for addictive behaviors, the authors of ACSID-11 stated that a larger database would be valuable not just for instrument testing but also for the entire field of research (2). The eight factors and the general domain are adequately represented across online pornography and dating applications used as a special form of social networking. It suggests that ACSID-11 adequately covers disorder-specific features in symptom manifestations. The data in our study represent English-speaking Internet users.

Further investigation among patients with diagnosed specific Internet-use disorders could be worth testing. Furthermore, exploring the impact of sexual orientation and relationship status in this area could yield interesting findings, as existing literature suggests that both variables may play a significant role in the use of online pornography and dating application profiles (42, 43). In our study, we observed that variables such as sexual orientation and relationship status may have important implications for understanding the context in which specific Internet-use behaviors manifest. For instance, individuals with a non-heterosexual orientation may engage with dating apps or online pornography in ways shaped by minority stress, identity exploration, or limited access to offline dating opportunities (44). Beyond relationship status, a range of psychological and contextual factors—such as use-related motivations, craving intensity, cue reactivity, loneliness, social stigma, and perceived social support—may also influence patterns of engagement with these platforms and the risk of developing problematic use (45). These factors highlight the importance of adopting a multidimensional framework when investigating behavioral addictions. Moreover, only 0.90% of respondents in our sample identified as non-binary, underscoring the need to validate the questionnaire across gender-diverse populations. Gender identity can shape online sexual behaviors, access to digital services, and vulnerability to harm, thereby influencing the development and expression of addictive patterns (46).

Another limitation is that the current sample included only individuals who reported using both online pornography and Tinder. While this approach ensured that participants could respond to both ASCID-11 versions, it also offered the advantage of evaluating distinct digital behaviors within the same individuals—an important strength given that most users engage with multiple online services. This design supports the assessment of the scale’s capacity to differentiate between behaviors while controlling for individual differences. However, it limits the ability to determine whether the factorial structure and psychometric properties would generalize to individuals who use only one of these platforms. Future research should therefore consider separate validation studies within single-platform user groups.

## 5 Conclusions

The results of the study suggest that the ACSID-11 is a suitable tool for testing other (potential) specified disorders due to addictive behaviors, including Tinder-use and online pornography-use disorders, based on ICD-11 diagnostic criteria. It highlights the recognition that specific digital behavior can become a significant health problem. Moreover, the tool is also valid for English-speaking respondents. While not a diagnostic tool in itself, it can be valuable in assisting with screening, assessment, and follow-up of people and patients with online pornography use or Tinder use. Specifically, it can help clinicians in the early identification of individuals at risk of problematic digital behaviors through symptom screening, and in the evaluation of symptom severity. The scale can also be used to support clinical interviews, tailor psychoeducation, and monitor treatment progress. Future studies could also incorporate the scale into intervention research, enabling the assessment of treatment efficacy and changes in symptomatology over time.

## Data Availability

The raw data supporting the conclusions of this article will be made available by the authors, without undue reservation.

## Appendix 1

Socio-demographic questions and their corresponding response option

1. Gender

a. Male

b. Female

c. Non/binary – other

2. What year were you born?

3. Marital status

a. Single

b. Married

c. In a relationship

d. Divorced

e. Widowed

4. In total, how many years have you been in school? Including all school levels – from primary school to university or any higher education institution.

5. Indicate which of three following socio-economic level do you feel belong to?

a. Low socio-economic level

b. Intermediate socio-economic level

c. High socio-economic level

Four additional questions, titled: “In the last month, how much time have you spend on a typical week-day/week-end on Tinder/pornography?” were multiple-choice questions ranging from 1.

Open Science

We report how we determined our sample size, all data exclusions, all data inclusion/exclusion criteria, whether inclusion/exclusion criteria were established prior to data analysis, all measures in the study, and all analyses including all tested models. If we use inferential tests, we report exact p values, effect sizes, and 95% confidence or credible intervals.

Open Data: The data is available on request from the authors.

Open Materials: I confirm that there is sufficient information for an independent researcher to reproduce all of the reported methodology.

Preregistration of Studies and Analysis Plans: This study was not preregistered.

Open Analytic Code: The data is available on request from the authors.
